# *Trichoderma*: A Treasure House of Structurally Diverse Secondary Metabolites With Medicinal Importance

**DOI:** 10.3389/fmicb.2021.723828

**Published:** 2021-07-23

**Authors:** Jian-Long Zhang, Wen-Li Tang, Qing-Rong Huang, You-Zhi Li, Mao-Lian Wei, Lin-Lin Jiang, Chong Liu, Xin Yu, Hong-Wei Zhu, Guo-Zhong Chen, Xing-Xiao Zhang

**Affiliations:** ^1^School of Life Sciences, Ludong University, Yantai, China; ^2^Shandong Provincial Key Laboratory of Quality Safty Monitoring and Risk Assessment for Animal Products, Jinan, China; ^3^Shandong Aquaculture Environmental Control Engineering Laboratory, Yantai, China; ^4^Yantai Key Laboratory of Animal Pathogenetic Microbiology and Immunology, Yantai, China; ^5^Yantai Research Institute for Replacing Old Growth Drivers with New Ones, Yantai, China

**Keywords:** *Trichoderma*, secondary metabolites, chemical diversity, biological activity, bioactive compounds

## Abstract

Fungi play an irreplaceable role in drug discovery in the course of human history, as they possess unique abilities to synthesize diverse specialized metabolites with significant medicinal potential. *Trichoderma* are well-studied filamentous fungi generally observed in nature, which are widely marketed as biocontrol agents. The secondary metabolites produced by *Trichoderma* have gained extensive attention since they possess attractive chemical structures with remarkable biological activities. A large number of metabolites have been isolated from *Trichoderma* species in recent years. A previous review by Reino et al. summarized 186 compounds isolated from *Trichoderma* as well as their biological activities up to 2008. To update the relevant list of reviews of secondary metabolites produced from *Trichoderma* sp., we provide a comprehensive overview in regard to the newly described metabolites of *Trichoderma* from the beginning of 2009 to the end of 2020, with emphasis on their chemistry and various bioactivities. A total of 203 compounds with considerable bioactivities are included in this review, which is worth expecting for the discovery of new drug leads and agrochemicals in the foreseeable future. Moreover, new strategies for discovering secondary metabolites of *Trichoderma* in recent years are also discussed herein.

## Introduction

*Trichoderma* is a fungal genus that was first described in 1794 (Persoon, 1794). This genus is well adapted to various ecological niches and is ubiquitous in most types of soils, roots, and foliar environments. *Trichoderma* species are beneficial for their commercial enzymes, plant growth-accelerating abilities, and biocontrol of plant diseases, indicating their promising industrial, agricultural, and medicinal potential (Cai et al., 2013; Bhardwaj and Kumar, 2017; McMullin et al., 2017). Globally, *Trichoderma* has proved to achieve great success as effective biological control drugs (Keswani et al., 2014). Many *Trichoderma* species, such as *T. harzianum*, *T. hamatum*, *T. asperellum*, *T. atroviride*, *T. koningii*, *T. virens*, and *T. viride*, are lucratively used as potent biocontrol agents worldwide (Bhardwaj and Kumar, 2017). These fungal species exhibit outstanding biocontrol capability against pathogenic microorganisms either through indirect (scrambling for nutrients, changing the ambient conditions, stimulating plant growth and defense responses) or direct (mycoparasitism) mechanisms (Bhardwaj and Kumar, 2017). Moreover, in addition to ecological effects, it is well known that *Trichoderma* can produce secondary metabolites that not only participate in signal transduction but also go through communications with various organisms (Keswani et al., 2014; Zeilinger et al., 2016). It is also believed that the successes of *Trichoderma* as biocontrol drugs are, at least partially, due to their capacity to secrete abundant secondary metabolites (Zeilinger et al., 2016).

Fungi play an irreplaceable role in the drug discovery in the course of human history, as they possess unique abilities to synthesize diverse secondary metabolites with significant medical potential (Li X.Q. et al., 2020). The discovery of penicillin from the filamentous fungal species *Penicillium* was a milestone in pharmaceutical research (Fleming, 1929). Since then, chemical studies regarding fungal secondary metabolites have become a research hotspot (Zhang P. et al., 2020). A large number of fungal secondary metabolites have been discovered, many of which have potential as drug leads (Newman and Cragg, 2016). The fungal species belonging to *Penicillium* and *Talaromyces* are representative flora, with many secondary metabolites possessing intriguing chemical skeletons and bioactivities characterized from these species (Frisvad, 2014). For the genus *Trichoderma*, more than 1000 metabolites have been isolated from *Trichoderma* in recent years (Zeilinger et al., 2016). Accordingly, many reviews on various aspects of *Trichoderma*, not only for the chemical diversity of metabolites but also for the various bioactivities and their potential applications, have been published. Zeilinger et al. (2016) reviewed the selected *Trichoderma*-derived secondary metabolites and gave an all-round summary of genomic analysis and putative gene clusters involved in biosynthesis. Keswani et al. (2014) listed targeted metabolites of *Trichoderma* and pointed out the utilization potentiality in multifarious areas, especially in agriculture. As mentioned above, *Trichoderma* is a well-known biocontrol agent that is used globally. Since many *Trichoderma* species are some of the most prominent producers of anti-phytopathogenic secondary metabolites, Khan et al. (2020) exhibited 45 fungicidal secondary metabolites of *Trichoderma* sp. along with the structural overview, biosynthesis pathway, and action mechanism. Moreover, Reino et al. (2008) systematically summarized the metabolites of *Trichoderma* and their bioactivities. As of 2008, a total of 186 compounds and 269 references were cited, including a detailed study of the activities of biocontrol mechanisms (Reino et al., 2008). Herein, in order to update the relevant list of reviews of secondary metabolites of *Trichoderma* sp., we provide a comprehensive overview in regard to the newly described metabolites of *Trichoderma* from the beginning of 2009 to the end of 2020, with emphasis on their chemistry and various bioactivities. Moreover, new strategies for discovering secondary metabolites of *Trichoderma* in recent years have also been discussed.

## Literature Search

To retrieve literature published up to 2020, an in-depth inspection was performed (Zhang P. et al., 2020). The key words of “*Trichoderma*” and “secondary metabolites” were used to search for related literatures in Web of Science, with the times pan from 2009 to 2020 (Supplementary Figure 1). Additionally, other platforms such as Crossref, Google Scholar, Elsevier, and Springer Link were also searched at the same time. The retrieved articles were categorized according to natural product chemistry. In all, 63 records in the context of natural product research were retained and assessed to present this review. A total of 203 compounds were found from *Trichoderma* from 2009 to 2020. It should be pointed out that some omissions were inevitable during the literature search. However, with the greatest effort, we have demonstrated almost all of the relevant research herein.

## Chemical Diversity

### Terpenoids

#### Trichothecene Sesquiterpenes

**FIGURE 1:**
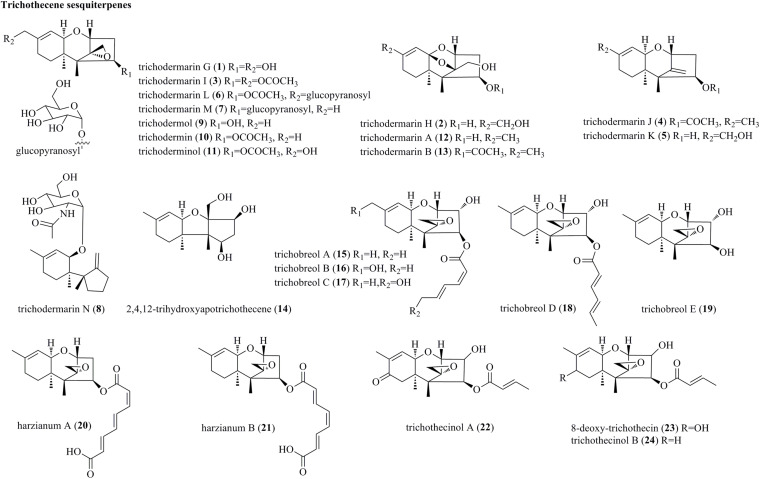
Trichothecene sesquiterpenes produced by *Trichoderma* species (**1–24**).

Trichothecenes, which are primarily produced by several genera of fungi, are sesquiterpene-based compounds possessing a tricyclic 12,13-epoxytrichothec-9-ene skeleton (Takahashi-Ando et al., 2020). Structurally, trichothecenes are classified into different families of nivalenols, neosolaniols, isotrichodermins, calonectrins, trichothecene, and trichobreols based on the substitution pattern. To date, more than 200 trichothecene derivatives have been discovered (Shi Z.Z. et al., 2020; Takahashi-Ando et al., 2020). The structures of trichothecenes isolated from *Trichoderma* species are listed in Figure 1. Eight newly discovered trichothecenes, trichodermarins G–N (**1**–**8**), and six known trichothecenes, trichodermol (**9**), trichodermin (**10**), trichoderminol (**11**), trichodermarin A (**12**) and trichodermarin B (**13**), and 2,4,12-trihydroxyapotrichothecene (**14**), were isolated from *Trichoderma brevicompactum* ADL-9-2, which was obtained as an endophyte of marine algae *Chondria tenuissima* (Shi Z.Z. et al., 2020). Trichodermarin N (**8**), featuring a 2’-*N*-acetylglucosaminyl moiety, represented the first aminoglycoside-bearing trichothecene. Chemical investigations of the marine fungus *Trichoderma* cf. *brevicompactum* TPU199 fermented with NaI afforded three new trichothecenes, trichobreols A–C (**15**–**17**) (Yamazaki et al., 2020a). Interestingly, **15** can be produced under the original culture conditions (seawater medium), whereas compounds **16** and **17** were found only under NaI-containing culture conditions. Additionally, isolation of the same fungus yielded two new trichothecenes, trichobreols D (**18**) and E (**19**) (Yamazaki et al., 2020b). Harzianums A (**20**) and B (**21**), two trichothecenes linked with octa-2,4,6-trienedioyl moiety, were isolated from the biofertilizer fungus *T. brevicompactum* (CGMCC19618) (Yin et al., 2020). From a marine fungus *Trichoderma longibrachiatum*, three trichothecenes, trichothecinol A (**22**), 8-deoxy-trichothecin (**23**), and trichothecinol B (**24**) were yielded (Du et al., 2020).

#### Carotane Sesquiterpenes

**FIGURE 2:**
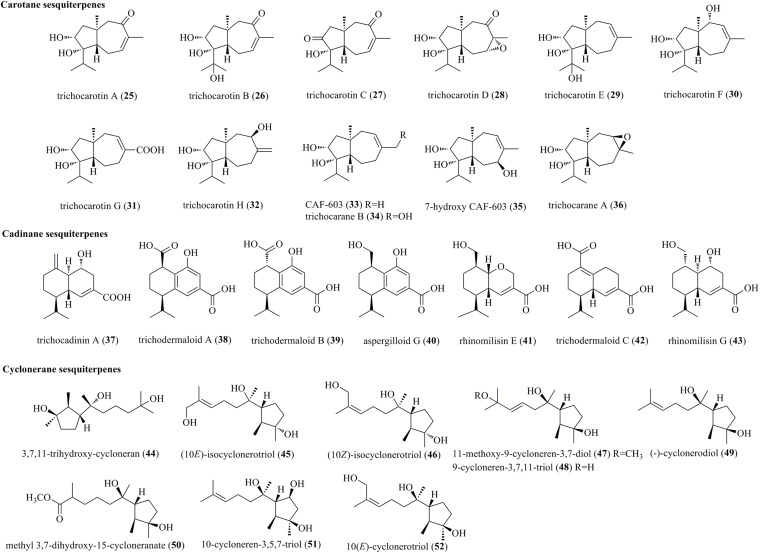
Carotane, cadinane, and cyclonerane sesquiterpenes produced by *Trichoderma* species (**25–52**).

From the marine-derived fungus *T. virens* Y13-3, eight undescribed carotane sesquiterpenes, trichocarotins A–H (**25**–**32**), along with the known compounds CAF-603 (**33**), trichocarane B (**34**), 7-β-hydroxy CAF-603 (**35**), and trichocarane A (**36**), were discovered (Figure 2) (Shi et al., 2018a). Carotane sesquiterpenes are commonly found in plants. However, only approximately 10 compounds have been isolated from filamentous fungi, representing a rare class of fungal metabolites (Shi et al., 2018a).

#### Cadinane Sesquiterpenes

A new example of a cadinane-skeletoned sesquiterpene, trichocadinin A (**37**), containing a previously unrecognized site of an exocyclic olefin functionality at C-10, was obtained from the marine-derived fungus *T. virens* Y13-3 (Shi et al., 2018a). This is the first time to report cadinane sesquiterpenes from *Trichoderma*. three new cadinane-type sesquiterpenes, i.e., trichodermaloids A–C (**38**–**40**), and three known ones, i.e., aspergilloid G (**41**), rhinomilisin E (**42**), and rhinomilisin G (**43**), were characterized from the marine sponge-derived fungus *Trichoderma* sp. SM16 (Cui et al., 2020).

#### Cyclonerane Sesquiterpenes

Induced by a chemical epigenetic manipulation strategy, a new cyclonerane, 3,7,11-trihydroxy-cycloneran (**44**), was produced by the marine fungus *T. harzianum* (XS-20090075), which was isolated from soft corals (Shi T. et al., 2020). Cycloneranes having a monocyclic skeleton are reported to be produced from various fungal genera, such as *Trichoderma*, *Aspergillus*, *Fusarium*, *Paecilomyces*, and *Trichothesium*. Based on previous findings, the five-membered ring of all the isolated cyclonerane sesquiterpenes featured the same relative configuration (Liu X.H. et al., 2020). However, chromatographic separation of the marine fungus *Trichoderma citrinoviride* A-WH-20-3 yielded two undescribed cycloneranes, (10*E*)-isocyclonerotriol (**45**) and (10*Z*)-isocyclonerotriol (**46**), which were characterized as the first example with an isomerized ring in cycloneranes (Liu X.H. et al., 2020). New cycloneranes, 11-methoxy-9-cycloneren-3,7-diol (**47**), methyl 3,7-dihydroxy-15-cycloneranate (**50**), and 10-cycloneren-3,5,7-triol (**51**), as well as two structurally related cycloneranes, 9-cycloneren-3,7,11-triol (**48**) and (–)-cyclonerodiol (**49**), were obtained from *T. harzianum* X-5, an endophyte of the marine alga *Laminaria japonica* (Song et al., 2018). Biosynthetically, **47** and **50** may be produced through *O*-methylation during the fermentation process. Two previously reported cycloneranes, i.e., 10-cycloneren-3,5,7-triol (**51**) and 10(*E*)-cyclonerotriol (**52**), were obtained from *T. longibrachiatum*, an endophyte of the highly halophile *Suaeda glauca* (Du et al., 2020).

#### Drimane Sesquiterpenes

**FIGURE 3:**
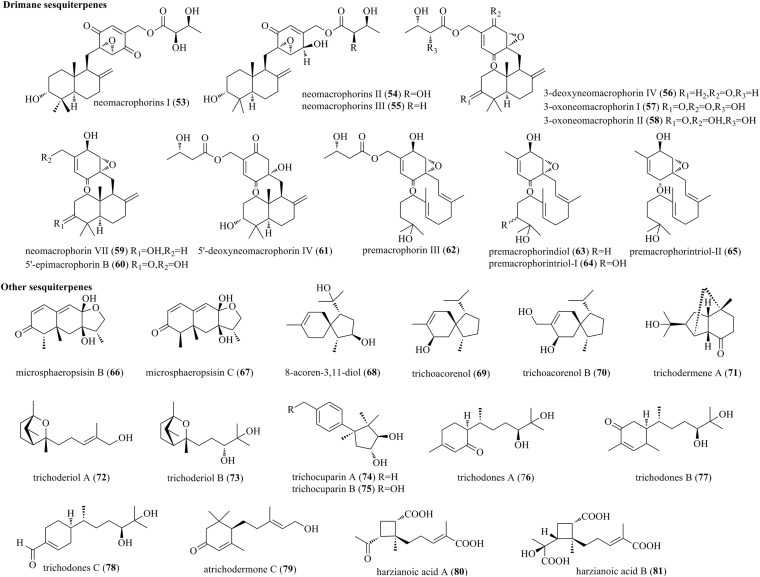
Drimane and other sesquiterpenes produced by *Trichoderma* species (**53–81**).

Chemical exploration of an endophyte *Trichoderma* sp. 1212-03 from *Daedaleopsis tricolor* yielded three new drimane sesquiterpenes, i.e., neomacrophorins I (**53**), II (**54**), and III (**55**) (Figure 3) (Hirose et al., 2014). They belong to macrophorins and drimane sesquiterpene-linked cyclohexenone epoxides but feature a hydroxyl in the drimene skeleton and a 5′,6′-α-epoxide in quinone functionality. Furthermore, six novel neomacrophorins, i.e., 3-deoxyneomacrophorin IV (**56**), 3-oxoneomacrophorins I (**57**) and II (**58**), neomacrophorin VII (**59**), 5′-epimacrophorin B (**60**), and 5′-deoxyneomacrophorin IV (**61**), as well as four novel premacrophorin congeners, i.e., premacrophorin III (**62**), premacrophorindiol (**63**), premacrophorintriols I (**64**), and II (**65**), were isolated from the same fungus (Nishiyama et al., 2019). These molecules possessed 2,3-epoxybenzoquinone (**56** and **57**) or 2,3-epoxybenzosemiquinol substructures (**58**–**60** and **62**–**64**). Premacrophorins **62**–**65** carried acyclic isoprenoid side chains biosynthetically derived from neomacrophorins in the early stage, rather than the common drimane skeleton.

#### Other Sesquiterpenes

Co-culture of the mangrove endophytic fungus *Trichoderma* sp. 307 and aquatic pathogenic bacterium *Acinetobacter johnsonii* B2 afforded two undescribed furan-type isoeremophilane sesquiterpenes, microsphaeropsisins B (**66**) and C (**67**) (Zhang et al., 2017). It is believed that both of them were derived from *Trichoderma* sp. rather than induced by the coculture. 8-Acoren-3,11-diol (**68**), trichoacorenol (**69**), and trichoacorenol B (**70**) were isolated from marine-derived *T. harzianum* X-5, and they were structurally characterized as acorane sesquiterpenes (Song et al., 2018). Trichodermene A (**71**), an unusual norsesquiterpene with a novel tricyclic-6/5/5-[4.3.1.0^1,6^]-decane framework, was characterized to originate from *T. longibrachiatum* (Du et al., 2020). Two new sesquiterpenes, i.e., trichoderiols A (**72**) and B (**73**), were produced by *T. atroviride* S361, an endophyte of *Cephalotaxus fortunei* (Zheng et al., 2011). Compounds **72** and **73** were structurally characterized as 2-oxabicyclo[2,1]heptane derivatives. New cuparenes, i.e., trichocuparins A (**74**) and B (**75**), were obtained from *T. brevicompactum* ADL-9-2, which was isolated from marine algae *C. tenuissima* (Shi Z.Z. et al., 2020). Compounds **74** and **75** are rare cuparenes with a cyclopentylcyclohexane unit. Three new sesquiterpenes, i.e., Trichodones A–C (**76**–**78**), were isolated from *T. asperellum* residing in *Panax notoginseng* (Ding et al., 2012a). The new sesquiterpenes possess the same skeleton as juvabione. However, the diol groups in compounds **76**–**78** were different from those of known juvabione analogs. Fermentation of *T. atroviride*, an endophyte of *Lycoris radiate*, afforded a new sesquiterpene, atrichodermone C (**79**) (Zhou et al., 2017). Chemical study of the marine sponge-associated fungus *T. harzianum* LZDX-32-08 yielded two new structurally unique sesquiterpenes, i.e., harzianoic acids A (**80**) and B (**81**) (Li et al., 2019). Interestingly, **80** and **81** had a cyclobutane framework, which was biogenetically derived from an unusual isoprenoid pathway.

#### Harziane Diterpenes

**FIGURE 4:**
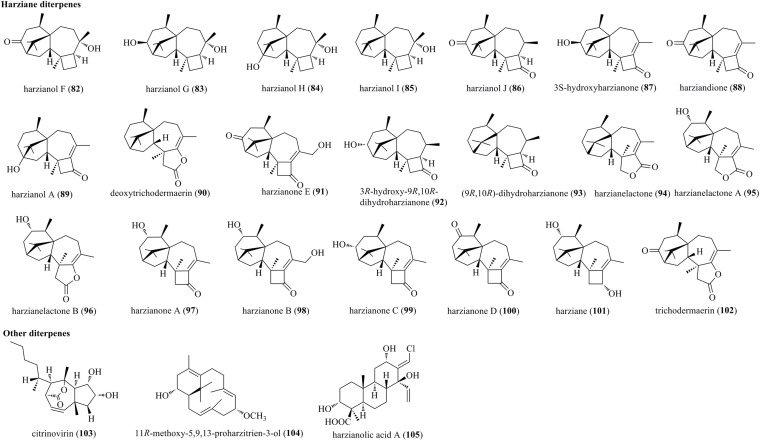
Harziane and other diterpenes produced by *Trichoderma* species (**82–105**).

Harziane diterpenes, typically possessing a 4/7/5/6-fused tetracyclic framework, have been reported exclusively from various *Trichoderma* species (Figure 4). Five undescribed harziane diterpenes, i.e., harzianols F–J (**82**–**86**), and three previously reported diterpenes, i.e., 3*S*-hydroxyharzianone (**87**), harziandione (**88**), and harzianol A (**89**), were isolated from the endophyte *T. atroviride* B7 of *Colquhounia coccinea* var. *mollis* (Li W.Y. et al., 2020). Deoxytrichodermaerin (**90**), an undescribed harziane lactone, was isolated from an endophyte *T. longibrachiatum* A-WH-20-2 of marine algae *Laurencia okamurai* (Zou et al., 2021). A new harziane diterpenoid, harzianone E (**91**), was obtained from the soft coral-sourced *T. harzianum* (XS-20090075) by chemical epigenetic manipulation strategy (Shi T. et al., 2020). A new harziane diterpene, 3*R*-hydroxy-9*R*,10*R*-dihydroharzianone (**92**), was produced by *T. harzianum* X-5 (Song et al., 2018). Two undescribed harzianes, i.e., (9*R*,10*R*)-dihydro-harzianone (**93**) and harzianelactone (**94**), were produced by *Trichoderma* sp. Xy24 from mangrove plant *Xylocarpus granatum* (Zhang et al., 2016). Chemical investigations on the marine fungus *T. harzianum* XS-20090075 yielded diverse harzianes, including two undescribed harziane lactones, i.e., harzianelactones A and B (**95** and **96**), as well as five new lactones, i.e., harzianones A–D (**97**–**100**) and harziane (**101**) (Zhao et al., 2019). Finally, a new diterpenoid lactone, trichodermaerin (**102**), was isolated from *Trichoderma erinaceum* derived from *Acanthaster planci* (Xie et al., 2013).

#### Other Diterpenes

Citrinovirin (**103**), a rare norditerpene, was produced by an endophyte *T. citrinoviride* cf-27 (Liang et al., 2016). Compound **103** possessed a perhydroazulene ring system, which was synthesized by a unique biogenetic pathway including demethylation, cyclization, oxidation, and S_*N*_2 reaction with Walden inversion. A new proharziane-type diterpene, 11*R*-methoxy-5,9,13-proharzitrien-3-ol (**104**), was characterized from the marine algicolous fungus *T. harzianum* X-5 (Song et al., 2018). **104** and harzianes were structurally related diterpenes. Harzianolic acid A (**105**), characterized as a novel chlorinated cleistanthane diterpenoid, was isolated from the marine fungal strain *T. harzianum* (XS-20090075) (Shi T. et al., 2020). Tricyclic diterpenoids categorized to cleistanthanes were reported from *Trichoderma* for the first time.

### Cyclopeptides

**FIGURE 5:**
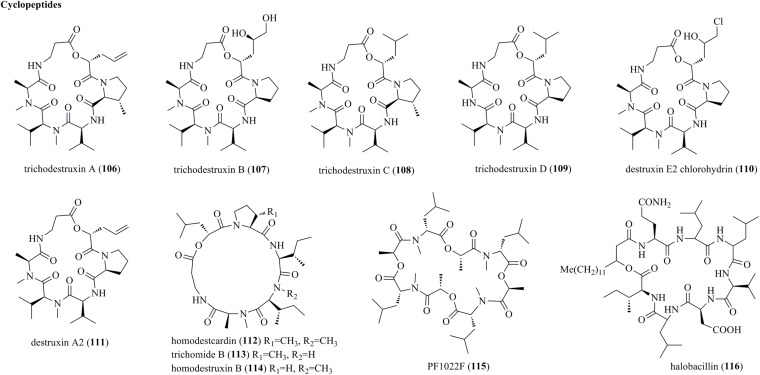
Cyclopeptides produced by *Trichoderma* species (**106–116**).

Bioassay-guided fractionation of the plant endophytic fungus *T. harzianum* KZ-20 afforded four new cyclodepsipeptides belonging to the destruxin family, i.e., trichodestruxins A–D (**106**–**109**), and two previously reported derivative, i.e., destruxin E2 chlorohydrin (**110**) and destruxin A2 (**111**) (Figure 5) (Liu Z. et al., 2020). Destruxins represent rare cyclic hexadepsipeptides. Structurally, new compound **107** possessed hydroxy acid fragments of the 2,4,5-trihydroxypentanoic acid unit, while **106** and **108** had a β-methylproline moiety. Homodestcardin (**112**), trichomide B (**113**), and homodestruxin B (**114**), characterized as cyclohexadepsipeptides of the trichomide series, were produced by *T. longibrachiatum* (Du et al., 2020). Finally, cyclopeptides PF1022F (**115**) and halobacillin (**116**) were obtained from the endophyte *T. asperellum* (Ding et al., 2012a).

### Diketopiperazines

**FIGURE 6:**
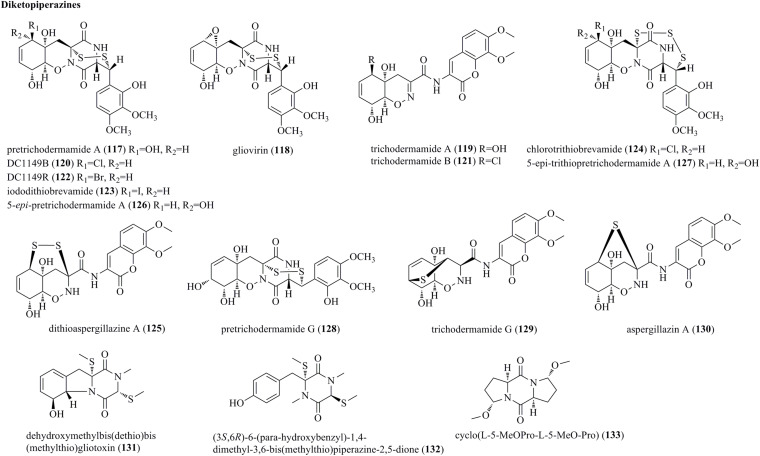
Diketopiperazines produced by *Trichoderma* species (**117–133**).

The marine fungus *Trichoderma* sp. TPU199 is a producer of a series of diketopiperazines (**117**–**127**) (Figure 6) (Yamazaki et al., 2020a). Initially, this fungal strain was found to produce pretrichodermamide A (**117**), gliovirin (**118**), and trichodermamide A (**119**). **117** and **118** possessed an unusual epipolythiodiketopiperazine (ETP) skeleton. Then, this strain with sodium halides added to the culture medium afforded the halogenated gliovirin-type ETPs DC1149B (**120**), DC1149R (**122**), and iododithiobrevamide (**123**). Subsequently, chlorotrithiobrevamide (**124**), the first trisulfide derivative in the type of ETP, was characterized. Furthermore, a highly modified dipeptide, dithioaspergillazine A (**125**), was obtained after the long time cultivation. Finally, two undescribed ETPs, i.e., 5-*epi*-pretrichodermamide A (**126**) and 5-*epi*-trithiopretrichodermamide A (**127**), were characterized under NaI-containing culture conditions. Pretrichodermamide G (**128**) was established as a 1,2-oxazadecaline ETP, and it was identified from the endophyte *T. harzianum* of *Zingiber officinale* (Harwoko et al., 2021). A rare heterocyclic dipeptide, i.e., trichodermamide G (**129**), and a biogenetically related metabolite aspergillazin A (**130**) were produced by the marine-sourced *T. harzianum* D13 (Zhao et al., 2020). Notably, **129** and **130** were novel ETP derivatives with the sulfur bridge locating at different positions. Dehydroxymethylbis(dethio)bis(methylthio)gliotoxin (**131**) and (3*S*,6*R*)-6-(para-hydroxybenzyl)-1,4-dimethyl-3,6-bis(methylthio)piperazine-2,5-dione (**132**), which were structurally characterized as two undescribed sulfurated diketopiperazines, were produced by an algicolous isolate of *T. virens* Y13-3 (Shi et al., 2018b). The fungal strain *T. asperellum* A-YMD-9-2 from *Gracilaria verrucosa* produced an undescribed symmetric diketopiperazine, cyclo(L-5-MeO-Pro-L-5-MeO-Pro) (**133**) (Song et al., 2020).

### Alkaloids and Other Nitrogen-Containing Compounds

**FIGURE 7:**
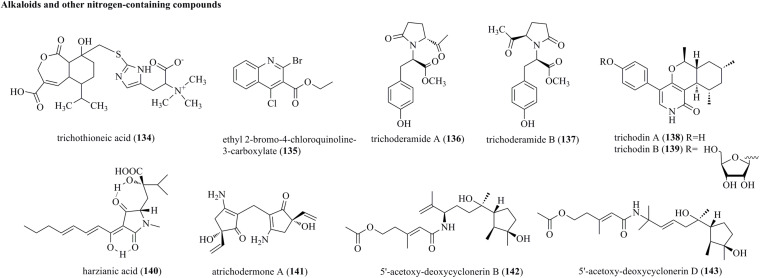
Alkaloids and other nitrogen-containing compounds produced by *Trichoderma* species (**134–143**).

Chemical survey of *T. virens* FKI-7573 generated an undescribed N-containing compound, i.e., trichothioneic acid (**134**) (Figure 7) (Miyano et al., 2020). **134** contained a heptelidic acid and an L-ergothioneine substructure. Ethyl 2-bromo-4-chloroquinoline-3-carboxylate (**135**) was produced by the soft coral-sourced *T. harzianum* (XS-20090075) in Czapek’s medium (Yu et al., 2021). **135** was the first halogenated quinoline derivative from *Trichoderma*. Trichoderamides A (**136**) and B (**137**), isolated as stereoisomers originating from the PKS-NRPS mixed pathway, were isolated from *T. gamsii*, an endophyte of *P. notoginseng* (Ding et al., 2015). Two rare pyridones, i.e., trichodins A (**138**) and B (**139**), were identified from *Trichoderma* sp. strain MF106 from the Greenland Seas (Wu et al., 2014). Harzianic acid (**140**), a nitrogen heterocyclic siderophore, was isolated from *T. harzianum* M10 (Vinale et al., 2013). **140** was identified as 2-hydroxy-2-[4-(1-hydroxy-octa-2,4-dienylidene)-1-methyl-3,5-dioxopyrrolidin-2-ylmethyl]-3-methyl-butyric acid. Atrichodermone A (**141**) was a unique compound with a dimeric cyclopentenone framework that was discovered from the endophytic fungal strain *T. atroviride* (Zhou et al., 2017). **141** was the first example of a 3-amino-5-hydroxy-5-vinyl-2-cyclopenten-1-one dimer. Two nitrogen-containing cyclonerane sesquiterpene derivatives, 5′-acetoxy-deoxycyclonerin B (**142**) and 5′-acetoxy-deoxycyclonerin D (**143**), were obtained from the marine fungus *T. asperellum* A-YMD-9-2 (Song et al., 2020).

### Polyketides

#### Naphthalene Derivatives

**FIGURE 8:**
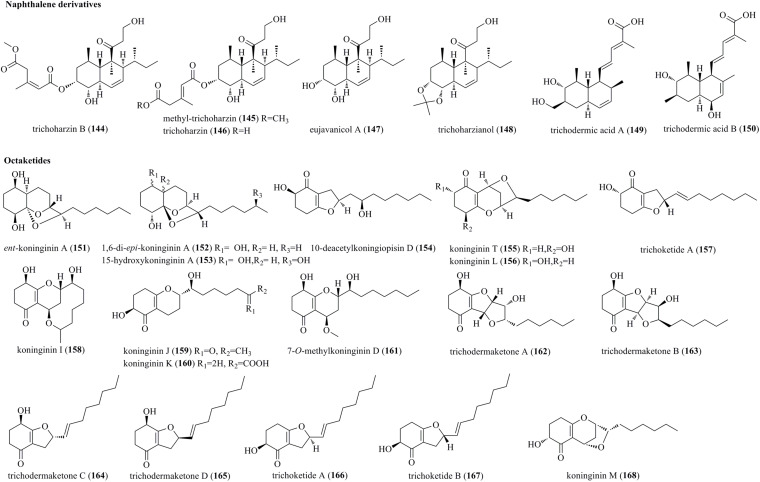
Naphthalene and octaketide derivatives produced by *Trichoderma* species (**144–168**).

An undescribed naphthalene, trichoharzin B (**144**), a natural product, methyl-trichoharzin (**145**), and the known trichoharzin (**146**) and eujavanicol A (**147**) were produced by the marine fungus *T. harzianum* XS-20090075 (Figure 8) (Yu et al., 2021). **144**–**146** were characterized as new polyketides with an alkylated decalin framework and esterified with a rare acyl group. Trichoharzianol (**148**) was identified from a fungal strain of *T. harzianum* F031 (Jeerapong et al., 2015). **148** was reported as a new decalin derivative bearing a 3-hydroxypropionyl moiety, a 1-methylpropyl moiety, and an acetonide moiety. Trichodermic acid A (**149**) and B (**150**) were isolated from an endophytic fungus *T. spirale*, and characterized as new octahydronaphthalene derivatives (Li et al., 2012).

#### Octaketides

Five new polyketides, *ent*-koninginin A (**151**), 1,6-di-*epi*-koninginin A (**152**), 15-hydroxykoninginin A (**153**), 10-deacetylkoningiopisin D (**154**), and koninginin T (**155**), along with two previously reported derivatives, koninginin L (**156**) and trichoketide A (**157**), were produced by *Trichoderma koningiopsis* QA-3 (Shi et al., 2017). Compounds **151**–**153** were characterized as tricyclic polyketides with an octahydrochromene skeleton. Koninginins I (**158**), J (**159**) and K (**160**), which were structurally characterized as new koninginin-type compounds, were produced by *T. neokongii* 8722 (Zhou et al., 2014). Chemical study of the marine-derived fungus *T. koningii* afforded five new polyketides, 7-*O*-methylkoninginin D (**161**) and trichodermaketones A–D (**162**–**165**) (Song et al., 2010). **162** and **163** represented unprecedented tricyclic polyketides having a bistetrafuran skeleton. Trichoketides A (**166**) and B (**167**), two undescribed octaketides, were isolated from *Trichoderma* sp. TPU1237 (Yamazaki et al., 2015). **166** and **167** were epimers at the α-position of the dihydrofuran ring. Finally, koninginins L (**156**) (herein reported as a new compound) and M (**168**) were isolated from solid fermentation of *T. koningii* 8662 (Lang et al., 2015). A series of koninginins were reported from *Trichoderma* species, but only in koninginins L (**156**) and M (**168**) was an oxygen bridge located between the C-10 and C-7 positions.

#### Cytochalasans

**FIGURE 9:**
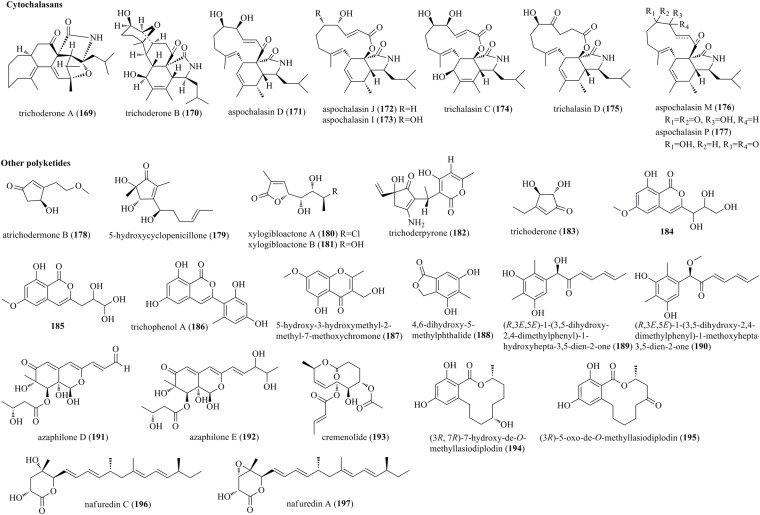
Cytochalasans and other polyketides produced by *Trichoderma* species (**169–197**).

Cytochalasans are a kind of novel fungal metabolic products, with more than 100 cytochalasans being reported to date. These metabolites contain a polycyclic skeleton and an isoindole moiety, which was fused with one macrocyclic ring (Ding et al., 2012c). Two highly complicated pentacyclic cytochalasans, trichoderone A (**169**) and trichoderone B (**170**), together with three previously reported cytochalasans, aspochalasins D (**171**), J (**172**), and I (**173**), were obtained from *T. gamsii* from *P. notoginseng* (Figure 9) (Ding et al., 2012c). **169** possessed a highly functionalized 7/6/6/5/5 pentacyclic skeleton, while **170** contained the unusual 6/5/6/6/5 pentacyclic framework. Furthermore, two undescribed cytochalasans, trichalasins C (**174**) and D (**175**), as well as three known cytochalasans, aspochalasins D (**171**), M (**176**), and P (**177**), were obtained from the abovementioned strain *T. gamsii* (Ding et al., 2012b).

#### Other Polyketides

An undescribed cyclopentenone, atrichodermone B (**178**), was isolated from endophytic *T. atroviride* (Zhou et al., 2017). A newly discovered cyclopentenone, 5-hydroxycyclopeni cillone (**179**), was produced by the marine-sourced *Trichoderma* sp. HPQJ-34 (Fang et al., 2017). **179** possessed a 3-substituted 4,5-dihydroxy-2,5-dimethylcyclopent-2-enone skeleton. Two previously reported unsaturated lactones, xylogibloactones A (**180**) and B (**181**), were found from the marine fungus *T. harzianum* (XS-20090075) (Yu et al., 2021). Compounds **180** and **181** had a C_9_ polyketide framework with a γ-lactone moiety. Trichoderpyrone (**182**), a novel cyclopentenone-pyrone mixed polyketide, was produced by *T. gamsii* (Chen et al., 2017). **182** had two individual ring systems, i.e., a 3-aminocyclopent-2-*en*-1-one moiety and a 4-hydroxy-6-methyl-2*H*-pyran-2-one moiety, which were derived from a mixed biosynthetic pathway. Trichoderone (**183**), a new cyclopentenone, was obtained from the marine *Trichoderma* sp. (You et al., 2010). Compounds **184** and **185**, characterized as two new isocoumarin derivatives with a butanetriol residue, were isolated from the endophytic fungus *T. harzianum* Fes1712 (Ding et al., 2019). Trichophenol A (**186**), which was identified as a new isocoumarin derivative with a 6,8-dihydroxyisocoumarin moiety, was isolated from *T. citrinoviride* A-WH-20-3 (Liu X.H. et al., 2020). Previously described metabolites, 5-hydroxy-3-hydroxymethyl-2-methyl-7-methoxychromone (**187**) and 4,6-dihydroxy-5-methylphthalide (**188**), were produced by the fungus *T. harzianum* F031 (Jeerapong et al., 2015). Two structurally new polyketides (**189** and **190**) with a 1-hydroxyhepta/methoxyhepta-3,5-dien-2-one moiety were produced in the transformant of *Trichoderma afroharzianum* (Ding et al., 2020). Two undescribed azaphilone derivatives, azaphilones D (**191**) and E (**192**), were obtained from dragonfly associated *T. harzianum* QTYC77 (Zhang S. et al., 2020). A new 10-membered lactone cremenolide (**193**), elucidated as but-2-enoic acid 7-acetoxy-6-hydroxy-2- methyl-10-oxo-5,6,7,8,9,10-hexahydro-2*H*-oxecin-5-yl ester, was isolated from cultural filtrates of *Trichoderma cremeum* (Vinale et al., 2016). Two undescribed polyketides, (3*R*,7*R*)-7-hydroxy-de-*O*-methyllasiodiplodin (**194**) and (3*R*)-5-oxo-de-*O*-methyllasiodiplodin (**195**), were isolated from the cocultivation of *Trichoderma* sp. 307 and *A. johnsonii* B2 (Zhang et al., 2017). An undescribed polyketide, nafuredin C (**196**), and the known nafuredin A (**197**), were produced by marine fungus *T. harzianum* D13 (Zhao et al., 2020).

### Other Compounds

**FIGURE 10:**
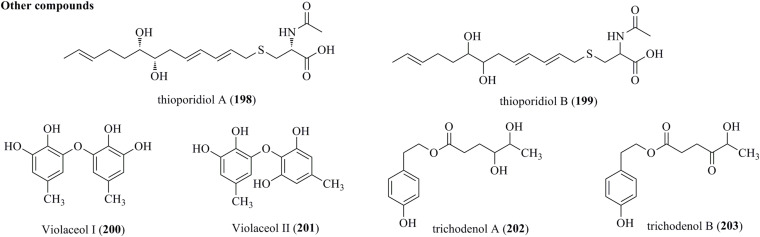
Other compounds produced by *Trichoderma* species (**198–203**).

Two new sulfur compounds, designated thioporidiols A (**198**) and B (**199**), were produced by a culture broth of *T. polypori* FKI-7382 (Figure 10) (Matsuo et al., 2020). Both of them were determined to be C13 lipid structures with an *N*-acetylcysteine moiety. A chemical investigation of the endophytic fungus *T. polyalthiae* offered two diphenyl ethers, Violaceol I (**200**) and II (**201**). Notably, both of them were characterized from *Trichoderma* for the first time (Nuankeaw et al., 2020). Trichodenols A (**202**) and B (**203**), two new compounds with 4-(2-hydroxyethyl) phenol moieties, were isolated from an endophyte *T. gamsii* (Ding et al., 2015).

## New Strategies for Discovering Secondary Metabolites of *Trichoderma*

**FIGURE 11:**
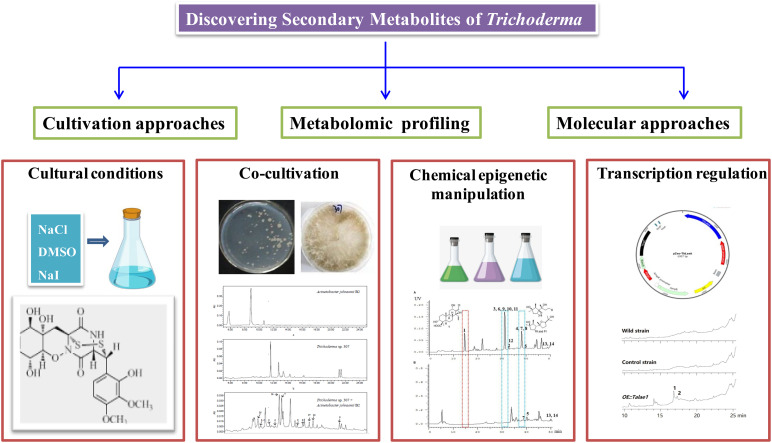
New strategies for discovering secondary metabolites of *Trichoderma.*

Recent fungal genome sequencing indicated that the majority of biosynthetic gene clusters (BGCs) associated with secondary metabolites are cryptic (transcriptionally silent) or expressed at very low levels under general laboratory conditions (Ren et al., 2017). Therefore, despite a large number of secondary metabolites being characterized from *Trichoderma*, genome sequencing revealed that there were more BGCs than we discovered, especially in filamentous fungi. These findings suggested that those silent metabolic pathways urgently need to be stimulated, which may lead to the discovery of novel metabolites with attractive functions. To activate cryptic biosynthetic pathways, many innovative approaches, such as cultivation-based approaches, metabolomic profiling, and genome mining-based molecular approaches, have been developed in recent years. These new approaches were accomplished with various degrees of success. The following are typical examples of searching for secondary metabolites of *Trichoderma* induced by cultivation regulation, cocultivation, chemical epigenetic manipulation, and transcript regulation, as shown in Figure 11.

**FIGURE 12:**
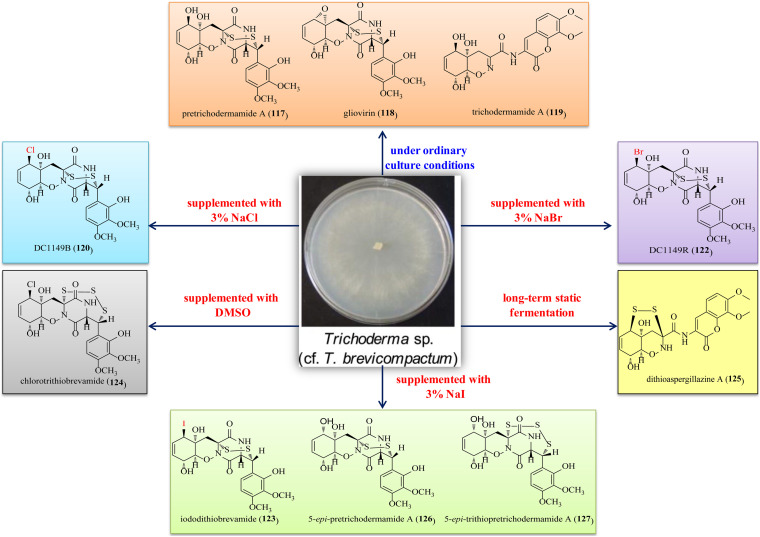
Diketopiperazines produced by *Trichoderma* sp. TPU199 under different conditions.

The marine-derived fungus *Trichoderma* sp. TPU199 was found to produce a series of diketopiperazines under different conditions (Figure 12) (Yamazaki et al., 2020a). Chemical investigations of this fungal strain under ordinary culture conditions led to the discovery of compounds **169**–**177**. Then, this fungus produced the halogenated gliovirin-type ETPs **120** (Cl derivative of **117**), **122** (Br derivative of **117**), and **123** (I derivative of **177**) when added with NaCl, NaBr, and NaI in culture medium, respectively. Moreover, TPU199 supplemented with DMSO yielded **124**, a new trithio derivative of **120**. A continuous study indicated that, with the long time cultivation, an undescribed modified dipeptide **125** was obtained. Finally, two undescribed ETPs **126** and **127** were characterized under NaI-containing culture conditions. It is undoubtedly proven that changing the culture conditions can activate cryptic metabolic pathways.

Microorganism coculture based on microbial interspecies competition is an efficient path to stimulate cryptic BGCs. Cocultivation of *Trichoderma* sp. 307 and pathogenic bacterium *A. johnsonii* B2 yielded two undescribed sesquiterpenes (**66** and **67**) and de-*O*-methyllasiodiplodin (**194** and **195**) (Zhang et al., 2017). HPLC analysis indicated that they were derived from *Trichoderma* sp. rather than by the coculture. Chemical epigenetic manipulation has also proven to be an effective method to activate cryptic BGCs. Therefore, it was applied to the marine fungal strain *T. harzianum* XS-20090075 to mine its potential to synthesize secondary metabolites (Shi T. et al., 2020). A histone deacetylase inhibitor, sodium butyrate at 10 μM, significantly changed its metabolic profile and gave rise to three undescribed terpenoids, including a cleistanthane **105**, a harziane diterpenoid **91**, and a cyclonerane sesquiterpenoid **44**. Interestingly, harziane diterpenoids were the dominant metabolites from this fungal strain under ordinary culture conditions, indicating the production of harzianes as the dominant metabolic pathway. In this study, the production of harzianes was hampered due to chemical epigenetic manipulation. In contrast, the biosynthetic pathways of cleistanthanes and cycloneranes were successfully activated, leading to the isolation of the new cleistanthane diterpenoid **105** and a series of cyclonerane sesquiterpenoids (**44** and other known cycloneranes). This is the first report of cleistanthane diterpenoids isolated from *Trichoderma* species. This study provided solid example to show that it is efficient to activate the silent genes of *Trichoderma* species by chemical epigenetic manipulation.

The transcriptional control has also proven to be an effective approach. To activate the chemical potential of the endophytic fungus *T. afroharzianum*, a *laeA*-like gene overexpression transformant was built (Ding et al., 2020). Further chemical investigation of this transformant successfully yielded two new antifungal polyketides (**189** and **190**). This study indicated that transcriptional control could be a considerable strategy in activating more secondary metabolites and enhancing the silent potential metabolism of *Trichoderma* species.

## Biological Activities

The producing fungus, environmental source, and bioactivities of compounds **1**–**203** are listed in Table 1. As shown in Table 1, most compounds possess various moderate to potent biological activities. Among them, antimicrobial, antimicroalgal, and anticancer activities represent dominant bioactivities to assess the pharmacological potential of these natural products. Detailed descriptions of these metabolites with promising biological activities are described as follows.

**TABLE 1:** The producing strain, environmental source, and biological activities of compounds **1**–**203**.

Compounds	Producing Strain	Environment Source	Biological Activities	References
Trichodermarins G–N (**1**–**8**), trichodermol (**9**), trichodermin (**10**), trichoderminol (**11**), trichodermarins A (**12**) and B (**13**), 2,4,12-trihydroxyapotrichothecene (**14**)	*T. brevicompactum* A-DL-9-2	Isolated from the marine red alga *Chondria tenuissima* collected from Dalian, China	Potent antifungal and antimicroalgal activities	Shi Z.Z. et al., 2020
Trichobreols A–C (**15**–**17**)	*T.* cf. *brevicompactum* TPU199	Isolated from an unidentified red alga, collected at the coral reef in Palau	Antifungal activity	Yamazaki et al., 2020a
Trichobreols D (**18**) and E (**19**)	*T.* cf. *brevicompactum* TPU199	Isolated from an unidentified red alga, collected at the coral reef in Palau	Antifungal activity	Yamazaki et al., 2020b
Harzianums A (**20**) and B (**21**)	*T. brevicompactum* (CGMCC19618)	Isolated from soil	Potent herbicidal activity	Yin et al., 2020
Trichothecinol A (**22**), 8-deoxy-trichothecin (**23**), trichothecinol B (**24**)	*T. longibrachiatum*	Isolated from the root of *Suaeda glauca*, a highly halophile plant	Potent antifungal activity	Du et al., 2020
Trichocarotins A–H (**25**–**32**), CAF-603 (**33**), trichocarane B (**34**), 7-β-hydroxy CAF-603 (**35**), trichocarane A (**36**), trichocadinin A (**37**)	*T. virens* Y13-3	Isolated from the surface of the marine red alga *Gracilaria vermiculophylla* collected from Yantai, China	Potent antimicroalgal activity	Shi et al., 2018a
Trichodermaloids A–C (**38**–**40**), aspergilloid G (**41**), rhinomilisin E (**42**), rhinomilisin G (**43**)	*Trichoderma* sp. SM 16	Isolated from a marine sponge *Dysidea* sp. collected from the Xisha Islands	Moderate anticancer activity	Cui et al., 2020
3,7,11-trihydroxy-cycloneran (**44**)	*T. harzianum* (XS-20090075)	Isolated from fresh tissue of an unidentified soft coral collected from Xisha Islands	No obvious antibacterial activity	Shi T. et al., 2020
(10*E*)-isocyclonerotriol (**45**), (10*Z*)-isocyclonerotriol (**46**)	*T. citrinoviride* A-WH-20-3	Isolated from the inner tissue of the red alga *Laurencia okamurai*	Moderate antimicroalgal activity	Liu X.H. et al., 2020
11-methoxy-9-cycloneren-3,7-diol (**47**), 9-cycloneren-3,7,11-triol (**48**), (–)-cyclonerodiol (**49**), methyl 3,7-dihydroxy-15-cycloneranate (**50**), 10-cycloneren-3,5,7-triol (**51**)	*T. harzianum* X-5	Isolated from the marine brown alga *Laminaria japonica*	Moderate to potent antimicroalgal activity	Song et al., 2018
10-cycloneren-3,5,7-triol (**51**), 10(*E*)-cyclonerotriol (**52**)	*T. longibrachiatum*	Isolated from the root of *Suaeda glauca*, a highly halophile plant	Moderate nematicidal activity	Du et al., 2020
Neomacrophorins I (**53**), II (**54**), III (**55**)	*Trichoderma* sp. 1212-03	Isolated from *Daedaleopsis tricolor* in Shirakami Mountains area	Moderate antifungal and anticancer activities	Hirose et al., 2014
3-deoxyneomacrophorin IV (**56**), 3-oxoneomacrophorin I (**57**), 3-oxoneomacrophorin II (**58**), neomacrophorin VII (**59**), 5′-epimacrophorin B (**60**), 5′-deoxyneomacrophorin IV (**61**), premacrophorin III (**62**), premacrophorindiol (**63**), premacrophorintriol-I (**64**), premacrophorintriol-II (**65**)	*Trichoderma* sp. 1212-03	Isolated from *Daedaleopsis tricolor* in Shirakami Mountains area	Moderate to potent anticancer activity	Nishiyama et al., 2019
Microsphaeropsisins B (**66**), C (**67**)	*Trichoderma* sp. 307	Isolated from the stem bark of mangrove *Clerodendrum inerme*	Weak α-glucosidase inhibitory activity	Zhang et al., 2017
8-acoren-3,11-diol (**68**), trichoacorenol (**69**), trichoacorenol B (**70**)	*T. harzianum* X-5	Isolated from the marine brown alga *L. japonica*	Moderate antimicroalgal activity	Song et al., 2018
Trichodermene A (**71**)	*T. longibrachiatum*	Isolated from the root of *Suaeda glauca*, a highly halophile plant	Potent antifungal activity	Du et al., 2020
Trichoderiols A (**72**), B (**73**)	*T. atroviride* S361	Isolated from the bark of *Cephalotaxus fortune*	Potent anti-inflammatory activity	Zheng et al., 2011
Trichocuparins A (**74**), B (**75**)	*T. brevicompactum* A-DL-9-2	Isolated from the marine red alga *Chondria tenuissima*	No obvious antifungal activity	Shi Z.Z. et al., 2020
Trichodones A (**76**), B (**77**), C (**78**)	*T. asperellum*	Isolated from the traditional Chinese medicinal plant *Panax notoginseng*	No obvious antibacterial activity	Ding et al., 2012a
Atrichodermone C (**79**)	*T. atroviride*	Isolated from the bulb of *Lycoris radiate*	No obvious anti-inflammatory and cytotoxic activities	Zhou et al., 2017
Harzianoic acids A (**80**), B (**81**)	*T. harzianum* LZDX-32-08	Isolated from the marine sponge *Xestospongia testudinaria*	Moderate anti-HCV activity	Li et al., 2019
Harzianols F–J (**82**–**86**), 3*S*-hydroxyharzianone (**87**), harziandione (**88**), harzianol A (**89**),	*T. atroviride* B7	Isolated from the healthy flowers of *Colquhounia coccinea* var. *mollis*	Potent antibacterial activity and moderate cytotoxicity	Li W.Y. et al., 2020
Deoxytrichodermaerin (**90**)	*T. longibrachiatum* A-WH-20-2	Isolated from marine red alga *L. okamurai*	Potent antimicroalgal activity	Zou et al., 2021
Harzianone E (**91**)	*T. harzianum* (XS-20090075)	Isolated from an unidentified soft coral	Weak antibacterial activity	Shi T. et al., 2020
3*R*-hydroxy-9*R*,10*R*-dihydroharzianone (**92**)	*T. harzianum* X-5	Isolated from the marine brown alga *L. japonica*	Moderate antimicroalgal activity	Song et al., 2018
(9*R*,10*R*)-dihydro-harzianone (**93**), harzianelactone (**94**)	*Trichoderma* sp. Xy24	Isolated from mangrove plant *Xylocarpus granatum*	Moderate anticancer activity	Zhang et al., 2016
Harzianelactones A, B (**95**, **96**), harzianones A–D (**97**–**100**), harziane (**101**)	*T. harzianum* XS-20090075	Isolated from the inner part of an unidentified soft coral	Potent phytotoxicity	Zhao et al., 2019
Trichodermaerin (**102**)	*T. erinaceum* 2011F1-1	Isolated from the inner tissue of the sea star *Acanthaster planci*	No cytotoxic activity	Xie et al., 2013
Citrinovirin (**103**)	*T. citrinoviride* cf-27	Isolated from the marine brown alga	Moderate antibacterial activity	Liang et al., 2016
11*R*-methoxy-5,9,13-proharzitrien-3-ol (**104**)	*T. harzianum* X-5	Isolated from the marine brown alga *L. japonica*	Potent antimicroalgal activity	Song et al., 2018
Harzianolic acid A (**105**)	*T. harzianum* (XS-20090075)	Isolated from an unidentified soft coral	No antibacterial activity	Shi T. et al., 2020
Trichodestruxins A–D (**106**–**109**), destruxin E2 chlorohydrin (**110**), destruxin A2 (**111**)	*T. harzianum* KZ-20	Isolated from the inner tissue of fruit of *Physalis angulata* L.	Moderate to potent anticancer activity	Liu Z. et al., 2020
Homodestcardin (**112**), trichomide B (**113**), homodestruxin B (**114**)	*T. longibrachiatum*	Isolated from the root of *Suaeda glauca*	Moderate nematicidal activity	Du et al., 2020
Cyclopeptides PF1022F (**115**), halobacillin (**116**)	*T. asperellum*	Isolated from *P. notoginseng*	Weak antibacterial activity	Ding et al., 2012a
Pretrichodermamide A (**117**), gliovirin (**118**), trichodermamide A (**119**), DC1149B (**120**), DC1149R (**122**), iododithiobrevamide (**123**), chlorotrithiobrevamide (**124**), dithioaspergillazine A (**125**), 5-*epi*-pretrichodermamide A (**126**), 5-*epi*-trithiopretrichodermamide A (**127**)	*T.* cf. *brevicompactum* TPU199	Isolated from an unidentified red alga, collected at the coral reef in Palau	Untested activity	Yamazaki et al., 2020a
Pretrichodermamide G (**128**)	*T. harzianum*	Isolated from the medicinal plant *Zingiber officinale*	No obvious activity	Harwoko et al., 2021
Trichodermamide G (**129**), aspergillazin A (**130**)	*T. harzianum* D13	Isolated from the mangrove plant *Excoecaria agallocha*	Untested activity	Zhao et al., 2020
Dehydroxymethylbis(dethio)bis(methylthio) gliotoxin (**131**), (3*S*,6*R*)-6-(para-hydroxybenzyl)-1,4-dimethyl-3,6-bis(methylthio)piperazine-2,5-dione (**132**)	*T. virens* Y13-3	Isolated from the surface of the marine red alga *G vermiculophylla*	No obvious activity	Shi et al., 2018b
Cyclo(L-5-MeO-Pro-L-5-MeO-Pro) (**133**)	*T. asperellum* A-YMD-9-2	Isolated from the marine marcroalga *Gracilaria verrucosa*	No obvious activity	Song et al., 2020
Trichothioneic acid (**134**)	*T. virens* FKI-7573	Isolated from a soil sample collected in Obihiro, Japan	Potent antioxidant activity	Miyano et al., 2020
Ethyl 2-bromo-4-chloroquinoline-3-carboxylate (**135**)	*T. harzianum* (XS-20090075)	Isolated from an unidentified soft coral from the Xisha Islands	No obvious activity	Yu et al., 2021
Trichoderamides A (**136**) and B (**137**)	*T. gamsii*	Isolated from the traditional Chinese medicinal plant *P. notoginseng*	No obvious cytotoxic activity	Ding et al., 2015
Trichodins A (**138**) and B (**139**)	*Trichoderma* sp. Strain MF106	Isolated from a Greenland Sea (Fram Strait) sample	Moderate antimicrobial activity	Wu et al., 2014
Harzianic acid (**140**)	*T. harzianum* M10	Source was not given	Potent plant growth promotion activity	Vinale et al., 2013
Atrichodermone A (**141**)	*T. atroviride*	Isolated from the bulb of *L. radiate*	No obvious anti-inflammatory and cytotoxic activities	Zhou et al., 2017
5′-acetoxy-deoxycyclonerin B (**142**), 5′-acetoxy-deoxycyclonerin D (**143**)	*T. asperellum* A-YMD-9-2	Isolated from the marine marcroalga *G. verrucosa*	No obvious activity	Song et al., 2020
Trichoharzin B (**144**), methyl-trichoharzin (**145**), trichoharzin (**146**), eujavanicol A (**147**)	*T. harzianum* (XS-20090075)	Isolated from an unidentified soft coral from the Xisha Islands	Moderate antifouling activity	Yu et al., 2021
Trichoharzianol (**148**)	*T. harzianum* F031	Isolated from soil collected in Suphanburi, Thailand	Moderate antifungal activity	Jeerapong et al., 2015
Trichodermic acid A (**149**), trichodermic acid B (**150**)	*T. spirale*	Isolated from the medicinal plant *Aquilaria sinensis*	Untested activity	Li et al., 2012
*ent*-koninginin A (**151**), 1,6-di-*epi*-koninginin A (**152**), 15-hydroxykoninginin A (**153**), 10-deacetylkoningiopisin D (**154**), koninginin T (**155**), koninginin L (**156**), trichoketide A (**157**)	*T. koningiopsis* QA-3	Isolated from the inner tissue of *Artemisia argyi* that was collected from Qichun, China	Moderate antibacterial activity	Shi et al., 2017
Koninginins I (**158**), J (**159**) and K (**160**)	*T. neokongii* 8722	Source was not given	No obvious antifungal activity	Zhou et al., 2014
7-*O*-methylkoninginin D (**161**), trichodermaketones A–D (**162**–**165**)	*T. koningii*	Isolated from marine mud of the South China Sea	No obvious antifungal activity	Song et al., 2010
Trichoketides A (**166**) and B (**167**)	*Trichoderma* sp. TPU1237	Isolated from a seawater sample collected at Aomori, Japan	Moderate PTP1B inhibitory activity	Yamazaki et al., 2015
Koninginins L (**156**) and M (**168**)	*T. koningii* 8662	Source was not given	No obvious antifungal activity	Lang et al., 2015
Trichoderones A (**169**) and B (**170**), aspochalasins D (**171**), J (**172**), I (**173**)	*T. gamsii*	Isolated from the medicinal plant *P. notoginseng*	Moderate cytotoxic activity	Ding et al., 2012c
Trichalasins C (**174**), D (**175**), aspochalasins D (**171**), M (**176**), P (**177**)	*T. gamsii*	Isolated from the medicinal plant *P. notoginseng*	Moderate cytotoxic activity	Ding et al., 2012b
Atrichodermone B (**178**)	*T. atroviride*	Isolated from the bulb of *Lycoris radiate*	No obvious anti-inflammatory and cytotoxic activities	Zhou et al., 2017
5-hydroxycyclopeni cillone (**179**)	*Trichoderma* sp. HPQJ-34	Isolated from the sponge *Hymeniacidon perleve* collected from Dongji Island.	Moderate antioxidative, anti-Aβ fibrillization and neuroprotective activities	Fang et al., 2017
Xylogibloactones A (**180**) and B (**181**)	*T. harzianum* (XS-20090075)	Isolated from an unidentified soft coral from the Xisha Islands	No obvious activity	Yu et al., 2021
Trichoderpyrone (**182**)	*T. gamsii*	Isolated from the medicinal plant *P. notoginseng*	Weak cytotoxic activity	Chen et al., 2017
Trichoderone (**183**)	*Trichoderma* sp. GIBH-Mf082	Isolated from marine sediment in the South China Sea	Potent anticancer activity	You et al., 2010
**184** and **185**	*T. harzianum* Fes1712	Isolated from *Ficus elastica* leaves	Moderate antibacterial activity	Ding et al., 2019
Trichophenol A (**186**)	*T. citrinoviride* A-WH-20-3	Isolated from the red alga *L. okamurai*	Moderate antimicroalgal activity	Liu X.H. et al., 2020
5-hydroxy-3-hydroxymethyl-2-methyl-7-methoxychromone (**187**), 4,6-dihydroxy-5-methylphthalide (**188**)	*T. harzianum* F031	Isolated from soil collected in Suphanburi, Thailand	No obvious antifungal activity	Jeerapong et al., 2015
(*R*,3*E*,5*E*)-1-(3,5-dihydroxy-2,4-dimethylphenyl)-1-hydroxyhepta-3,5-dien-2-one (**189**), (*R*,*3E*,*5E*)-1-(3,5-dihydroxy-2,4-dimethylphenyl)-1-methoxyhepta-3,5-dien-2-one (**190**)	*T. afroharzianum* Fes1712	Isolated from fresh leaves of *F. elastica*	Moderate antifungal activity	Ding et al., 2020
Azaphilones D (**191**) and E (**192**)	*T. harzianum* QTYC77	Isolated from the intestine of *Pantala flavescens*	Moderate antibacterial activity	Zhang S. et al., 2020
Cremenolide (**193**)	*T. cremeum* 506	Isolated from decaying wood in Central Poland	Potent antifungal and plant growth promotion activity	Vinale et al., 2016
(3*R*,7*R*)-7-hydroxy-de-*O*-methyllasiodiplodin (**194**), (3*R*)-5-oxo-de-*O*-methyllasiodiplodin (**195**)	*Trichoderma* sp. 307	Isolated from the stem bark of mangrove *C. inerme*	Potent α-glucosidase inhibitory activity	Zhang et al., 2017
Nafuredin C (**196**), nafuredin A (**197**)	*T. harzianum* D13	Isolated from the mangrove plant *E. agallocha*	Moderate antifungal activity	Zhao et al., 2020
Thioporidiols A (**198**), B (**199**)	*T. polypori* FKI-7382	Isolated from a sediment sample collected at Omuta city	Moderate antibacterial activity	Matsuo et al., 2020
Violaceol I (**200**), Violaceol II (**201**)	*T. polyalthiae*	Source was not given	Moderate antimicrobial activity	Nuankeaw et al., 2020
Trichodenols A (**202**), B (**203**)	*T. gamsii*	Isolated from the traditional Chinese medicinal plant *P. notoginseng*	No obvious cytotoxic activity	Ding et al., 2015

*The bold values are compounds numbers.*

### Antimicrobial Activities

Isolated trichothecene derivatives **1**–**14** were assayed for antifungal activity against *Botrytis cinerea*, *Cochliobolus miyabeanus*, *Fusarium oxysporum* f. sp. *cucumerium*, *F. oxysporum* f. sp. *niveum*, and *Phomopsis asparagi* (Shi Z.Z. et al., 2020). Compounds **1**–**3** and **9**–**11** displayed promising antifungal activity with MICs of 4.0–64 μg/mL. Among them, **10** was the most active, while compounds **4**, **5**, **8**, and **12**–**14** were inactive. Structure–activity relationships (SAR) among these trichothecenes indicated that the acetoxy and methyl functionalities (compound **10**) were necessary, while the epoxide moiety and the ether linkage were other possibilities (Shi Z.Z. et al., 2020). Compounds **15**–**17** showed antifungal activities against *Candida albicans* and *Cryptococcus neoformans*, with MICs of 1.6–50 μg/mL (Yamazaki et al., 2020a). In the same way, compounds **18** and **19** were active, with MICs of 6.3, 12.5, and 25 μg/mL, respectively (Yamazaki et al., 2020b). Apparently, the diene group and 2’*Z*-configuration play an important role in antifungal activities. Trichothecenes **22** and **23** showed significant activities toward the soil-borne phytopathogens *Colletotrichum lagenarium* with an MIC value of 16 μg/mL, which was stronger than that of the positive control carbendazim (MIC, 32 μg/mL) (Du et al., 2020). Furthermore, both of them showed potency against carbendazim-resistant *B. cinerea*. In contrast, compared to those of **22** and **23**, trichothecene congener **24** only showed weak effects, indicating that the hydroxyl substituted in **23** may enhance its antifungal activity. Trichothecenes are reported to possess promising antifungal, phytotoxic and cytotoxic activities. *Trichoderma*-derived trichothecenes were mainly focused on their antifungal activity in the literature above, which highlighted their potential as biocontrol agents. Drimane sesquiterpenes **53**–**55** were active against *C. miyabeanus* by inducing hyphal branching at 1.0 and 10 μg/mL (Hirose et al., 2014). The novel norsesquiterpene **71** showed potent ability against *C. lagrnarium* with an MIC of 8 μg/mL (Du et al., 2020). The new harziane diterpene harzianol I (**85**) exhibited potent effect on *Staphylococcus aureus*, *Bacillus subtilis*, and *Micrococcus luteus*, with EC_50_s of 7.7, 7.7, and 9.9 μg/mL, respectively (Li W.Y. et al., 2020). It seemed that substitutions at C-2 and/or C-3 of harzianes may decrease their antibacterial activity. The novel norditerpene **103** inhibited *S. aureus* with an MIC of 12.4 μg/mL (Liang et al., 2016). Cyclopeptides **115** and **116** displayed weak ability against *Enterococcus faecium* with IC_50_s of 7.30 and 5.24 μM and against *S. aureus* with IC_50_s of 19.02 and 14.00 μM, respectively (Ding et al., 2012a). The new pyridine trichodin A (**138**) was active against *B. subtilis* (IC_50_, 27.05 μM), *Staphylococcus epidermidis* (24.28 μM), and *C. albicans* (25.38 μM) (Wu et al., 2014). Trichoharzianol (**148**) displayed mild activity against *Colletotrichum gloeosporioides*, with an MIC value of 128 μg/mL (Jeerapong et al., 2015). Polyketides **151** and **157** showed moderate activity against *Escherichia coli*, *Edwardsiella tarda*, *Vibrio anguillarum*, and *Vibrio parahaemolyticus*, with MICs of 8–64 μg/mL (Shi et al., 2017). Trichodermaketone A (**162**) was inactive against *C. albicans* (MIC > 125 μg/mL). However, it was active at 125 μg/mL when treated with 0.05 μg/mL ketoconazole (Song et al., 2010). New isocoumarins **184** and **185** exhibited growth inhibitory activity against *E. coli* with an MIC of 32 μg/mL (Ding et al., 2019). Polyketides **189** and **190** exhibited selective antifungal activity toward *B. cinerea*, *F. oxysporum*, and *C. lagenarium*, with MICs of 8–32 μg/mL (Ding et al., 2020). The new azaphilone **191** displayed moderate effect on *S. aureus* and *B. subtilis* with disc diameters of the zone of inhibition of 7.3 and 7.0 mm (Zhang S. et al., 2020). The new 10-membered lactone **193** significantly inhibited *F. oxysporum*, *B. cinerea*, and *Rhizoctonia solani* (Vinale et al., 2016). Nafuredins **196** and **197** showed strong activity against *Magnaporthe oryzae*, with MICs of 8.63 and 17.4 μM, respectively (Zhao et al., 2020).

### Antimicroalgal Activities

The antimicroalgal activity against marine phytoplankton (*Amphidinium carterae*, *Heterocapsa circularisquama*, *Heterosigma akashiwo*, and *Prorocentrum donghaiense*) of trichothecene derivatives **1**–**14** was evaluated. Notably, **10** featured the strongest effect, with IC_50_s of 1.7, 0.82, 0.91, and 1.4 μg/mL (Shi Z.Z. et al., 2020). Carotane sesquiterpenes **27**–**29**, **32**, and **36** exhibited strong activity against several phytoplankton, with IC_50_s of 0.24–1.2 μg/mL (Shi et al., 2018a). Proposed SAR study indicated that the carbonyl, hydroxyl, and the epoxy moiety play an important role in the antimicroalgal potency of carotenes. The cyclonerane sesquiterpene **45** was more active against *Karlodinium veneficum* than **46**, with an IC_50_ of 8.1 μg/mL (Liu X.H. et al., 2020). It is interesting that, compared to their isomerized derivatives (10*E*)- and (10*Z*)-cyclonerotriol, the isomerization of the five-membered ring greatly suppressed their antimicroalgal activities (Liu X.H. et al., 2020). Cycloneranes **47**–**51** exhibited moderate to potent growth inhibition against (*Chattonella marina*, *H. akashiwo*, *K. veneficum*, and *P. donghaiense* with low μg/mL range (Song et al., 2018). Compound **47** potently inhibited *C. marina* with an IC_50_ of 0.66 μg/mL. The new acorane sesquiterpene **68** exhibited mild growth inhibition of *C. marina* (IC_50_, 2.8 μg/mL), while the new proharziane diterpene **104** potently inhibited with IC_50_s of 1.2–4.3 μg/mL (Song et al., 2018). Additionally, the new harziane lactone **90** possessed potent activity, with IC_50_s of 0.53–2.7 μg/mL (Zou et al., 2021). The new isocoumarin **186** was active against *C. marina* (IC_50_, 4.4 μg/mL), *H. akashiwo* (9.1 μg/mL), and *P. donghaiense* (5.9 μg/mL) (Liu X.H. et al., 2020).

### Anticancer Activities

The cytotoxicities of cadinane sesquiterpenes **38**–**43** were evaluated against NCIH-460, NCI-H929, and SW620 cell lines (Cui et al., 2020). In contrast to the known compounds **41**–**43**, the newly reported sesquiterpenes **38**–**40** showed more potent cytotoxicities, with IC_50_s of 6.8–12.7 μM. Neomacrophorin I (**53**) showed cytotoxicity against human adenocarcinoma cells (COLO 201) with an IC_50_ of 46 μg/mL (Hirose et al., 2014). Neomacrophorins **56** and **61** were cytotoxic toward COLO 201 with IC_50_s of 20.5 and 18.2 μg/mL, respectively (Nishiyama et al., 2019). From a structural point of view, the common substructure of 2′-cyclohexene-1′,4′-dione was critical for cytotoxicity. The new harziane diterpene harzianol I (**85**) was observed to exhibit moderate cytotoxicity against NCI-H1975 (IC_50_, 58.72 μM), HepG2 (60.88 μM), and MCF-7 (53.92 μM) cell lines (Li W.Y. et al., 2020). **93** showed selective cytotoxicity toward HeLa and MCF-7, with IC_50_s of 30.1 and 30.7 μM, respectively (Zhang et al., 2016). Cyclodepsipeptides **106**–**111** were active on HT-29, A549, and P388, with IC_50_s of 0.7–19.1 μM (Liu Z. et al., 2020). Cytochalasans **171** and **172** showed cytotoxicity on HeLa with IC_50_s of 5.72 and 27.4 μM, respectively, whereas **169**, **170**, and **173**–**177** were inactive (IC_50_ > 40.0μM) (Ding et al., 2012b, c). The rare polyketide **182** displayed weak only cytotoxic activity toward A549 (IC_50_, 16.9 μM), HepG2 (30.8 μM), and HeLa (33.9 μM) (Chen et al., 2017). Cyclopentenone **183** displayed potent cytotoxicities against A549, NCI-H460, MCF-7, MDA-MB-435, HeLa, and DU-145, whereas it was inactive toward the normal human lung fibroblast cell line (You et al., 2010). The selectivity index was more than 100, which was even more remarkable than that of cisplatin.

### Phytotoxic Activities

In phytotoxicity assays, harzianums A (**20**) and B (**21**) decreased the shoot and root lengths of the dicot species *Brassica chinensis* and induced inhibitory effect of seed germination at 2 μg/mL (Yin et al., 2020). Moreover, **20** and **21** showed phytotoxicity against monocots, *Oryza sativa* and *Echinochloa crusgalli*, compared with the positive control 2,4-dichlorophenoxyacetic acid (a chlorophenoxy herbicide most commonly used worldwide). The results indicated that **20** and **21** possess potent herbicidal potential for dicotyledon and/or monocotyledon weeds. All of the isolated harzianes **95**–**101** exhibited potent phytotoxicity at 200 ppm (Zhao et al., 2019).

### Other Activities

Cyclonerane sesquiterpenes **51** and **52** exhibited certain nematicidal activity against *Meloidogyne incognita*, with second-stage juvenile (J2s) lethal rates of 38.2 and 42.7% at 200 μg/mL (Du et al., 2020). Cyclodepsipeptides **112**–**114** also showed nematicidal activity against *M. incognita* (Du et al., 2020). New sesquiterpenes **72** and **73** showed potent NO scavenging effects, with IC_50_s of 15.3 and 9.1 μM, respectively (Zheng et al., 2011). Harzianoic acids **80** and **81** exerted potency to decrease the HCV RNA with EC_50_s of 24.5 and 20.4 μM, respectively (Li et al., 2019). Trichothioneic acid (**134**) showed OH radical-scavenging and singlet oxygen-quenching ability in a dose-dependent manner, which was equivalent to those of positive controls (Miyano et al., 2020). The activity of harzianic acid (**140**) as a plant growth promoter was evaluated (Vinale et al., 2013). Treatment with 100 or 10 μM **140** significantly affected seed germination at 4 and 5 times stronger than that of the blank control. Naphthalene derivatives **145** and **147** showed moderate antifouling potency with EC_50_s of 29.8 and 35.6 μg/mL, respectively (Yu et al., 2021). The new de-*O*-methyllasiodiplodin **194** and **195** showed strong α-glucosidase inhibitory activity with IC_50_s of 25.8 and 54.6 μM, respectively, which were higher than acarbose (703.8 μM) (Zhang et al., 2017).

## Summary of the Studies

### Chemical Structures

**FIGURE 13:**
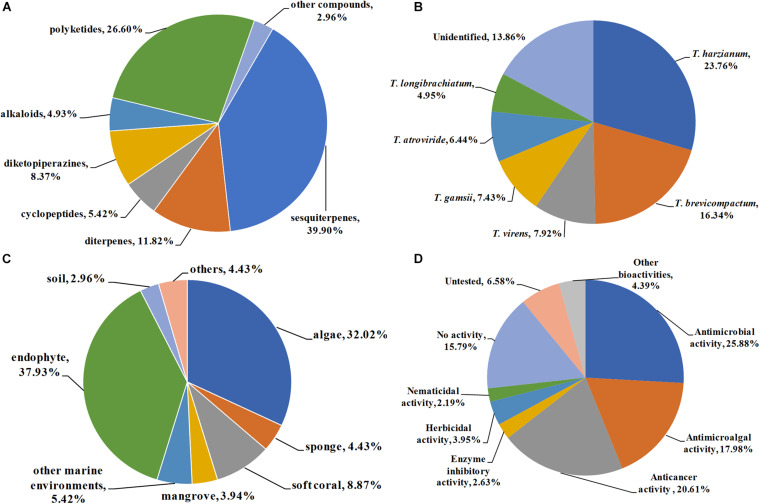
Quantification of this studies. **(A)** Chemical structures categories; **(B)** producing strains; **(C)** environment sources; and **(D)** bioactivity categories.

A total of 203 natural products were reported from *Trichoderma* from 2009–2020. Their chemical structures were classified into terpenoids (**1**–**24** for trichothecene sesquiterpenes, **25**–**36** for carotane sesquiterpenes, **37**–**43** for cadinane sesquiterpenes, **44**–**52** for cyclonerane sesquiterpenes, **53**–**65** for drimane sesquiterpenes, **66**–**81** for other sesquiterpenes, **82**–**102** for harziane diterpenes, and **103**–**105** for other diterpenes), cyclopeptides (**104**–**116**), diketopiperazines (**117**–**133**), alkaloids and other nitrogen-containing compounds (**134**–**143**), polyketides (**144**–**150** for naphthalene derivatives, **151**–**168** for octaketides, **169**–**177** for cytochalasans, and **178**–**197** for other polyketides), and other compounds (**198**–**203**) according to their putative biogenetic sources. As shown in Figure 13A, 39.9% of the metabolites reported were sesquiterpenes, followed by polyketides with 26.6%. Taking diterpenes into account, terpenoids accounted for 51.72% of the obtained compounds, which indicated that species belonging to *Trichoderma* are considerable producing strains of novel terpenoids. It should be pointed out that some terpenoids, such as harzianes, are isolated exclusively from *Trichoderma* species. This review described 21 harziane diterpenes produced by *Trichoderma*. Considering their intriguing structures and bioactivities, much more attention should be devoted to this type of terpenoid in subsequent chemical studies.

### Producing Strains

The genus *Trichoderma* comprises more than 340 species. Some of them are used as biocontrol agents, while some of them are promising producers of enzymes for industrial purposes. On the other hand, some *Trichoderma* species possess the unique capacity to synthesize various secondary metabolites with potent biological activities. In this review, a total of 17 identified species, including *T. harzianum*, *T. brevicompactum*, *T. virens*, *T. gamsii*, *T. atroviride*, *T. longibrachiatum*, *T. asperellum*, *T. koningiopsis*, *T. koningii*, *T. citrinoviride*, *T. neokongii*, *T. spirale*, *T. afroharzianum*, *T. polypore*, *T. polyalthiae*, *T. erinaceum*, and *T. cremeum*, are reported as the producing strains of the described metabolites. Among them, *T. harzianum* and *T. brevicompactum* were the most prolific strains, with 48 (23.76%) and 33 (16.34%) metabolites identified, respectively (Figure 13B). The fungus *T. harzianum* is famous for widely used biocontrol agents, and it is also considered to be a promising producer of bioactive metabolic products. *T. brevicompactum* can synthesize trichothecene-type sesquiterpenoids with potent antifungal activity and high biotechnological value. Twenty-one novel trichothecenes (**1**–**21**) have been characterized from *T. brevicompactum*.

### Environment Sources

The genus *Trichoderma* is widely distributed and has been isolated in soils, decaying wood, and endophytes in the inner tissue of host plants. Previous studies have mainly focused on terrestrial species of *Trichoderma*. However, *Trichoderma* from the marine environment are unexploited. It would be useful to examine marine-derived *Trichoderma* species since they may be induced to produce specific metabolites in hyperhaline environments. Accordingly, in recent years, increasing attention has been devoted to marine *Trichoderma*. As shown in Figure 13C, a total of 54.7% producing fungus were obtained from marine environments, including algae (32.02%), sponges (4.43%), soft corals (8.87), mangroves (3.94%), and other marine environments (5.42%, seawater, sediments), with 111 compounds characterized. Moreover, some fungi are obtained as endophytes from medicinal plants. Endophytic fungi, which harmoniously live in the inner tissues of their hosts without causing apparent diseases, are considered to be prolific sources of novel metabolites with remarkable pharmacological activities. It is estimated that 37.97% of these compounds were isolated from endophytic *Trichoderma*. From the above analysis, it can be concluded that marine environment and endophytes are more abundant sources of those productive strains.

### Biological Activities

As discussed above, most of the presented compounds possess considerable biological activities, such as antimicrobial, antimicroalgal, anticancer, enzyme inhibitory, herbicidal, and nematicidal activities. Among them, antimicrobial (25.88%), anticancer (20.61%), and antimicroalgal (17.98%) activities were dominant in assessing the pharmacological potential of these metabolites (Figure 13D). It should be pointed out that a high proportion (73.40%) of the presented metabolites showed moderate to potent bioactivities. Even more importantly, a large number of them exhibit potent activities, which are higher than those of positive controls. For example, trichothecenes **22** and **23** showed higher antifungal effect on *C. lagrnarium* (MIC, 16 μg/mL) than the synthetic fungicide carbendazim (MIC, 32 μg/mL) (Du et al., 2020). Cyclopentenone **183** displayed potent cytotoxicities, whereas it was inactive toward the normal lung cell line (You et al., 2010). The selectivity index was even more remarkable than that of cisplatin, indicating **183** has high selective toxicity to cancer cell lines. Sesquiterpenes **72** and **73** showed potent NO scavenging effects (Zheng et al., 2011). These impressive bioactivities indicate that many of these compounds could be used as potential candidates for new drug discovery.

## Conclusion

In the present review, we offer a detailed summary of recently isolated metabolites from *Trichoderma* from the beginning of 2009 to the end of 2020. As a result, a total of 203 metabolites are described herein, including their structural diversity and biological activities. Moreover, new strategies for discovering secondary metabolites of *Trichoderma* in recent years have also been discussed. *Trichoderma* has proven to be a treasure house of interesting secondary metabolites with medicinal importance. The biochemical studies of *Trichoderma* are untapped. Although a mass of metabolites have been isolated from *Trichoderma* species, the further excavation of those metabolites is worth expecting. By using new approaches to activate their silent gene clusters, including cultivation-based approaches, metabolomic profiling, and genome mining-based molecular approaches, an ever-increasing number of bioactive compounds will be obtained, which will be beneficial for the new drug discovery in the near future.

## Author Contributions

J-LZ and W-LT wrote this manuscript. Q-RH, Y-ZL, M-LW, L-LJ, CL, XY, H-WZ, and G-ZC collected and reorganized the literature data. X-XZ supervised the research work and revised the manuscript. All authors reviewed the manuscript.

## Conflict of Interest

The authors declare that the research was conducted in the absence of any commercial or financial relationships that could be construed as a potential conflict of interest.

## Publisher’s Note

All claims expressed in this article are solely those of the authors and do not necessarily represent those of their affiliated organizations, or those of the publisher, the editors and the reviewers. Any product that may be evaluated in this article, or claim that may be made by its manufacturer, is not guaranteed or endorsed by the publisher.
