# Supplementary material for: Trichoderma: A Treasure House of Structurally Diverse Secondary Metabolites With Medicinal Importance
Source: Front Microbiol. 2021 Jul 23;12:723828. doi: 10.3389/fmicb.2021.723828 (PMC8342961; doi:10.3389/fmicb.2021.723828)
Supplement: Supplementary file 1 [file Table_1.DOCX]

*Supplemental Material*

*Trichoderma*: A Treasure House of Structurally Diverse Secondary Metabolites with Medicinal Importance

Jian-Long Zhang ^1,2,4†^, Wen-Li Tang ^2,†^, Qing-Rong Huang ^1,3^, You-Zhi Li ^2^, Mao-Lian Wei ^2^, Lin-Lin Jiang ^1,2,4,5^, Chong Liu ^1^, Xin Yu ^1,2,3^, Hong-Wei Zhu ^1,2,4,5^, Guo-Zhong Chen ^1,3,4^, Xing-Xiao Zhang ^1,3,4,^*

^1^ School of Life Sciences, Ludong University, Yantai 264025, China

^2^ Shandong Provincial Key Laboratory of Quality Safty Monitoring and Risk Assessment for Animal Products, Ji'nan 250022, China

^3^ Yantai Key Laboratory of Animal Pathogenetic Microbiology and Immunology, Yantai 264025, China

^4^ Shandong Aquaculture Environmental Control Engineering Laboratory, Yantai 264000, Shandong, China

^5^ Yantai Research Institute for Replacing Old Growth Drivers with New Ones, Yantai 264000, Shandong, China

***Correspondence:**

Xing-Xiao Zhang

zhangxingxiao@ldu.edu.cn

^†^These authors contributed equally to this work.

Keywords: *Trichoderma*, secondary metabolites, chemical diversity, biological activity

**Figure S1.** The flowchart of literature search
